# Supplementary material for: Spectral preferences of mosquitos are altered by odors
Source: J Exp Biol. 2025 Jul 9;228(13):jeb250318. doi: 10.1242/jeb.250318 (PMC12276811; doi:10.1242/jeb.250318)
Supplement: Supplementary information [file jexbio-228-250318-s1.pdf]

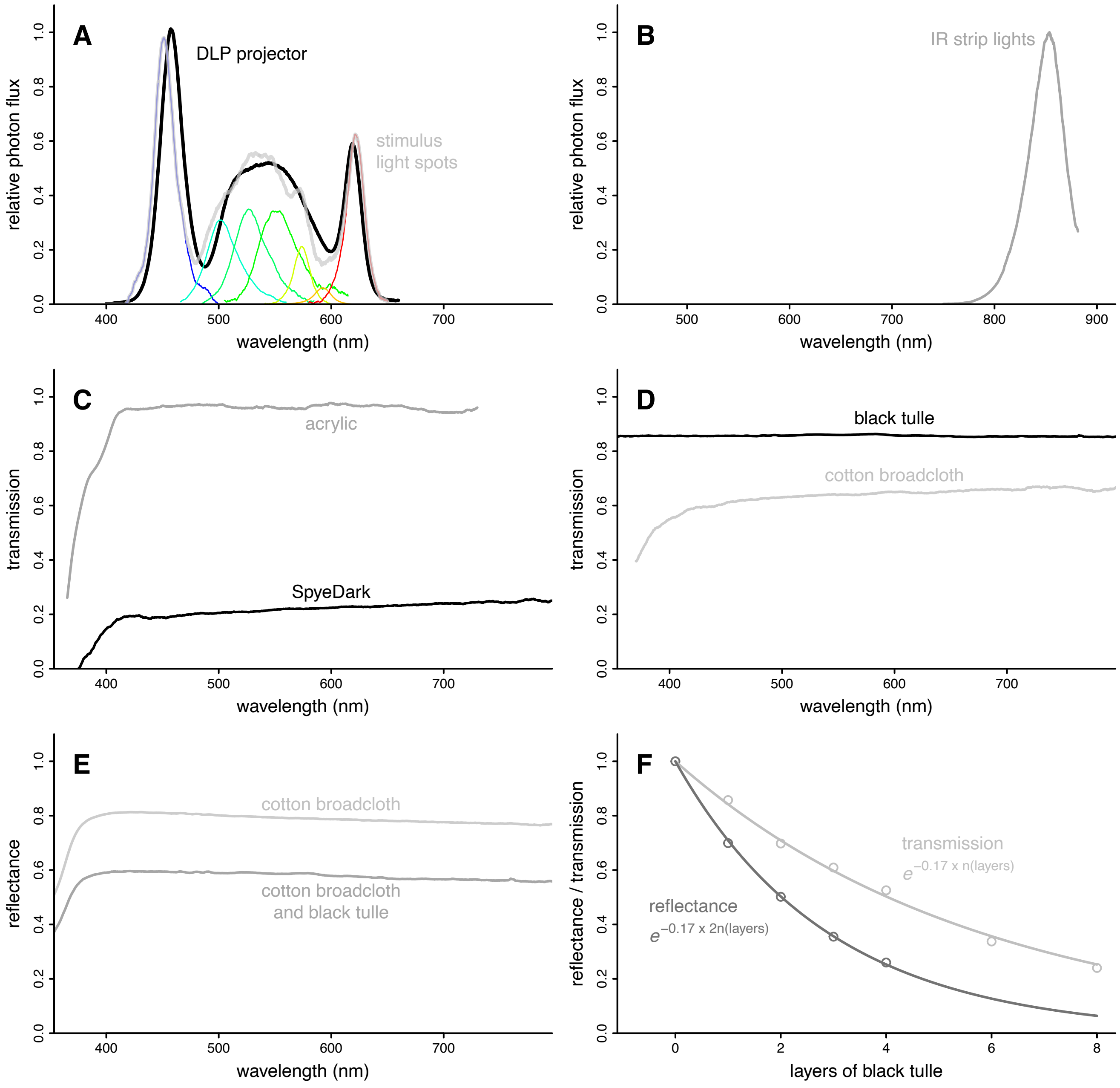

Fig. S1. Spectroscopy of surfaces and light sources used in the wind tunnel. (A) Relative photon flux of the DLP projectors providing ambient illumination in the wind tunnel and of the grayscale visual stimuli. The photon fluxes of the 7 component LED color channels are shown with the thinner colored lines below. (B) Relative photon flux of the IR LED strips used to provide illumination for the cameras.
(C) Transmission of acrylic walls and floor of the wind tunnel, and the SpyeDark panels lining the walls.
(D,E) Transmission and reflectance of the materials making up the fabric liner for the wind tunnel floor. The reflection of the cotton broadcloth and black tulle was estimated from the reflectance of cotton broadcloth corrected for transmission through two layers of black tulle (see S1f). (F) The relationship between transmission/reflectance and the number of tulle layers. Transmission measurements (light gray circles) estimated spectrographically, and reflectance measurements (dark gray circles) estimated photographically. Reflectance through a layer of tulle can be viewed as transmission through the layer by both the incident and reflected light.

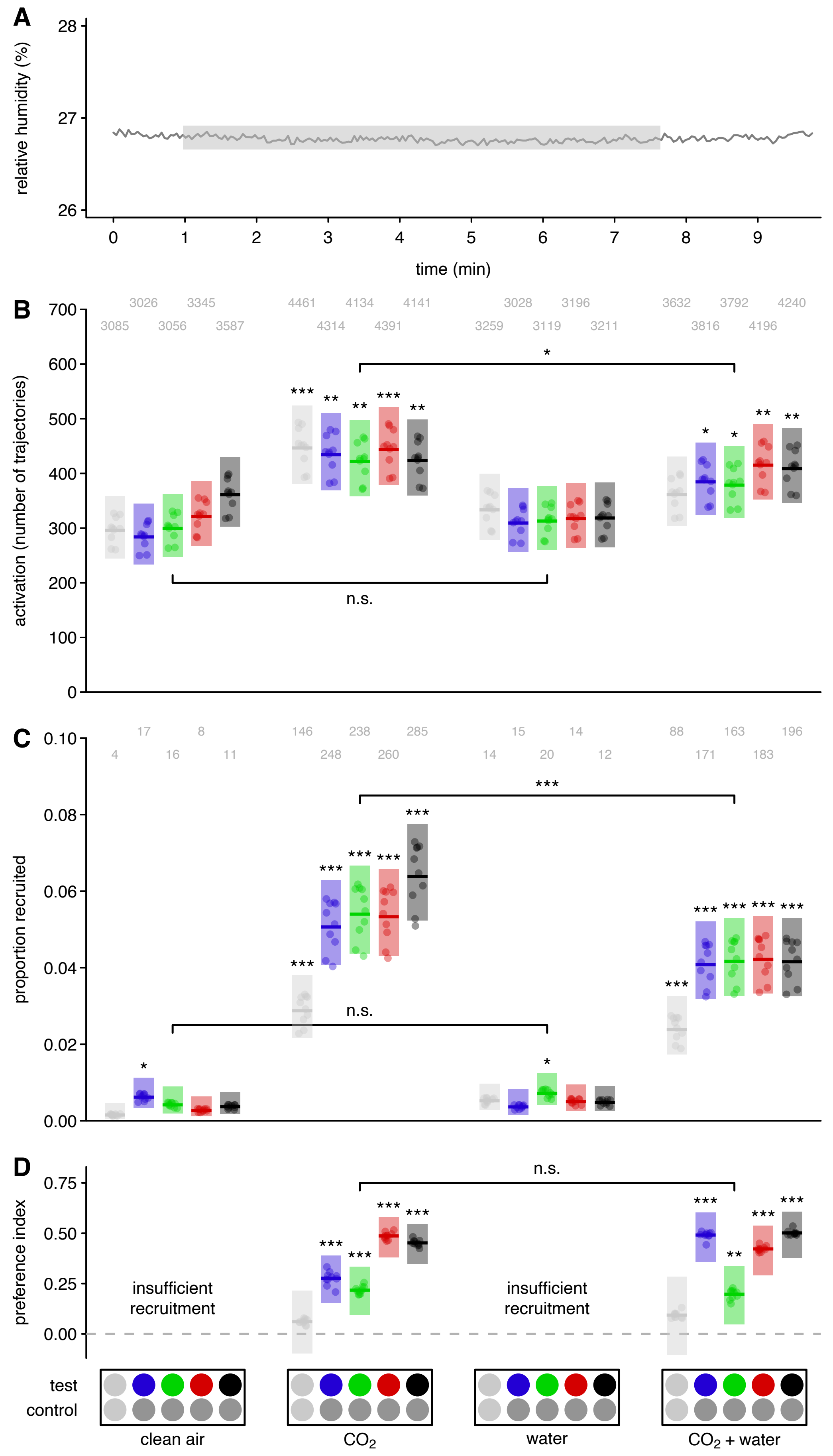

Fig. S2. Effect of humidity on mosquito behavior. (A) Relative humidity in the wind tunnel as measured by a sensor (SHT4x, Sensirion) located 10 cm downwind of the odor/CO_2 outlet. The gray box indicates the period where 10% of the plume air passed through the odor jar containing 100 ml of distilled water (humidified air), however no increase in humidity is apparent. (B) Mosquito activation as characterized by the number of mosquito trajectories recorded during each stimulus period where the plume consisted of clean air alone, 10% CO_2, humidified air, and the combination of CO_2 and humidified air. Asterisks above the boxes here indicate a statistical difference in the number of trajectories as compared with the stimulus period with paired neutral gray stimuli and clean air alone (leftmost box). When CO_2 was present, we found humidified air caused a significant decrease in the number of trajectories (significance bracket, *a priori* contrast, z = 2.16, P = 0.0310). Numbers above each boxplot indicate the total number of trajectories over 10 bioassay runs. (C) The proportion of trajectories recruited to either the test or control visual stimuli under the same odor conditions listed above. Asterisks above the boxes here indicate a statistical difference from the recruitment to paired neutral gray stimuli with clean air alone (leftmost box). When CO_2 was present, we found humidified air caused a significant decrease in recruitment (significance bracket, *a priori* contrast, z = 3.70, P = 0.0002). Numbers above each boxplot indicate the total number of recruited trajectories over 10 bioassay runs. (D) The preference index of mosquitos in the wind tunnel responding to visual stimuli of various wavelengths under the same odor conditions listed above. Asterisks above the boxes here indicate a statistical difference from a preference index of 0.00. We found no evidence that this humidified air had any effect of the visual preference of responding mosquitos (significance bracket, likelihood-ratio test, χ^2 = 6.96 df = 5 P = 0.22). Test stimuli from left to right: neutral gray (light gray circles) at an intensity matching the fabric background, blue (450 nm), green (527 nm), red (621 nm) LEDs at matching isoquantal intensities, and unilluminated black tulle targets (black circles). Control stimuli: neutral or mid gray. Boxplots are the mean (line) with 95% confidence interval (shaded area), with points representing model predictions for each replicate bioassay run. Asterisks denote statistical differences: n.s. > 0.05, *P < 0.05, **P < 0.01, ***P < 0.001

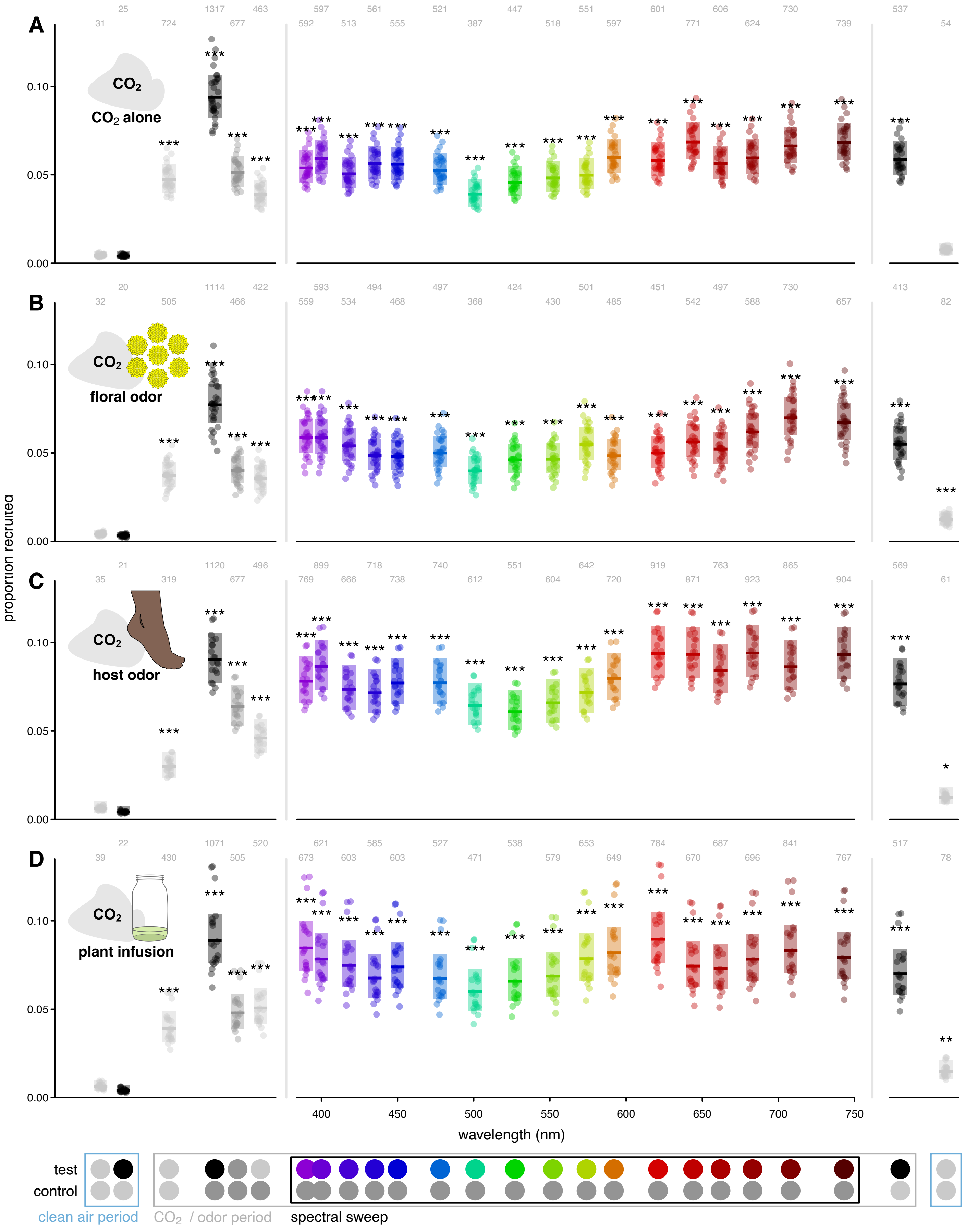

Fig. S3. Effect of odor on mosquito recruitment to visual stimuli during the spectral sweeps. Proportion of trajectories recruited to either the test or control visual stimulus in the presence of 10% CO_2 plume paired with (A) no odor, (B) tansy (*T. vulgare*) floral odor, (C) human foot odor, and (D) the odor of an alfalfa infusion. Test stimuli: neutral gray (light gray circles) at an intensity matching the fabric background, mid gray, unilluminated black tulle targets (black circles), and LEDs at isoquantal intensities ranging from 390-743 nm (Fig. 1B). Control stimuli: neutral or mid gray. Stimuli outside the marked CO_2 / odor period were presented with clean air alone. The order of the spectral sweep stimuli was alternated between bioassay runs, with all other stimuli pairs always appearing in the order depicted above. Boxplots are the mean (line) with 95% confidence interval (shaded area), with points representing model predictions for each replicate bioassay run. Numbers above each boxplot indicate the number of recruited trajectories over 30, 30, 20, and 20 replicate bioassay runs. Asterisks above the boxes indicate a statistical difference from the recruitment to paired neutral gray stimuli with clean air alone (leftmost box). Asterisks denote statistical differences: *$P < 0.05$, **$P < 0.01$, ***$P < 0.001$

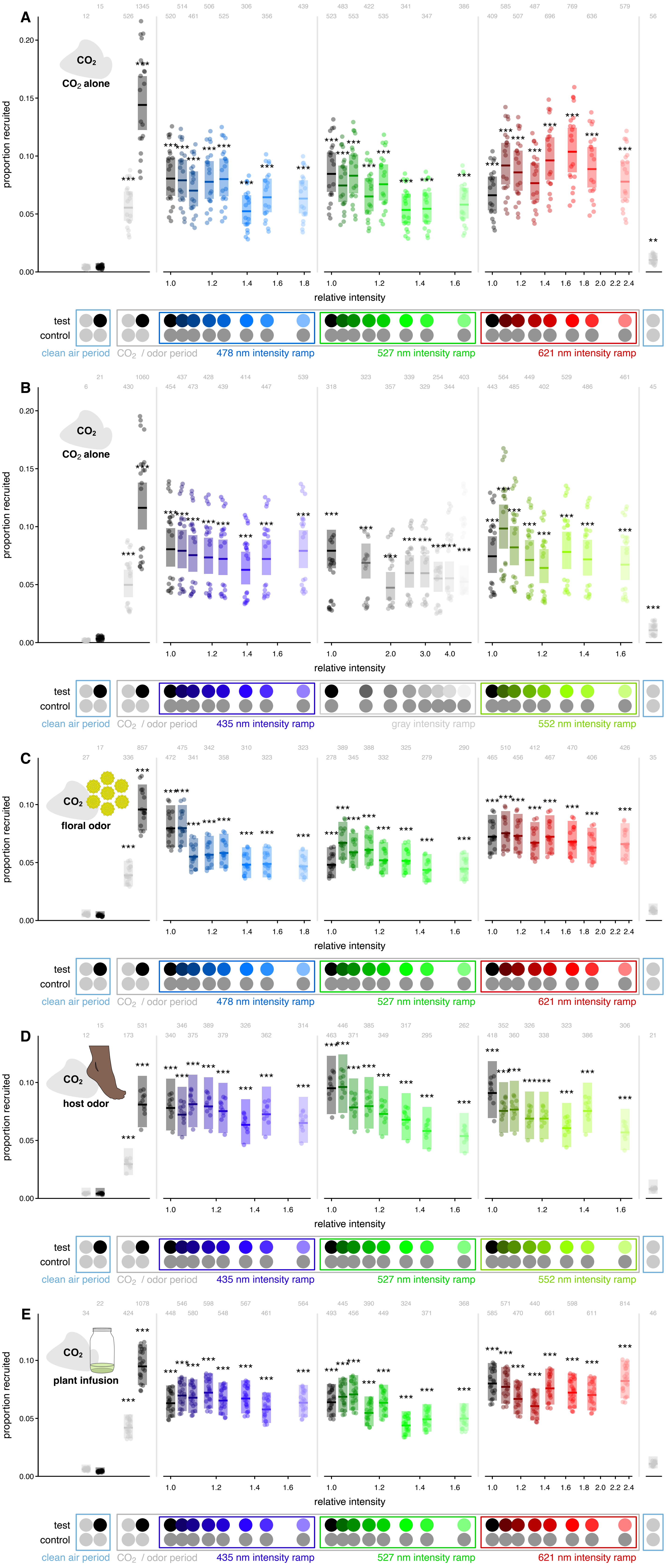

Fig. S4. Effect of odor and stimulus intensity on mosquito recruitment to visual stimuli. Proportion of trajectories recruited to either the test or control visual stimulus in the presence of a 10% CO_2 plume paired with (A,B) no odor, (C) tansy (*T. vulgare*) floral odor, (D) human foot odor, and (E) the odor of an alfalfa infusion. We investigated the effect of intensity at a selection of wavelengths covering the visible range and focusing on spectral ranges where we observed odor shifts in spectral preferences in the spectral sweep experiments. The intensities on the x-axes are measured relative to the unilluminated black tulle targets, which were common among all of the intensity ramps, and non-zero due to the ambient illumination. Test stimuli: neutral gray (light gray circles) at an intensity matching the fabric background, unilluminated black tulle targets (black circles), 435 nm, 478 nm, 527 nm, 552 nm, and 621 nm LED intensity ramps ranging in intensity from 0.0 to 3.0 times the isoquantal intensity used in the spectral sweep experiments, and a gray ramp ranging in intensity from 0.0 to 1.5 times the intensity of the fabric background. Control stimuli: neutral or mid gray. Stimuli outside the marked CO_2 / odor period were presented with clean air alone. Boxplots are the mean (line) with 95% confidence interval (shaded area), with points representing model predictions for each replicate bioassay run. Numbers above each boxplot indicate the number of recruited trajectories over 20, 20, 16, 10 and 20 replicate bioassay runs, respectively. Asterisks above the boxes denote a statistically significant difference from the recruitment to paired neutral gray stimuli with clean air alone (leftmost box). Asterisks denote statistical differences: *$P < 0.05$, **$P < 0.01$, ***$P < 0.001$

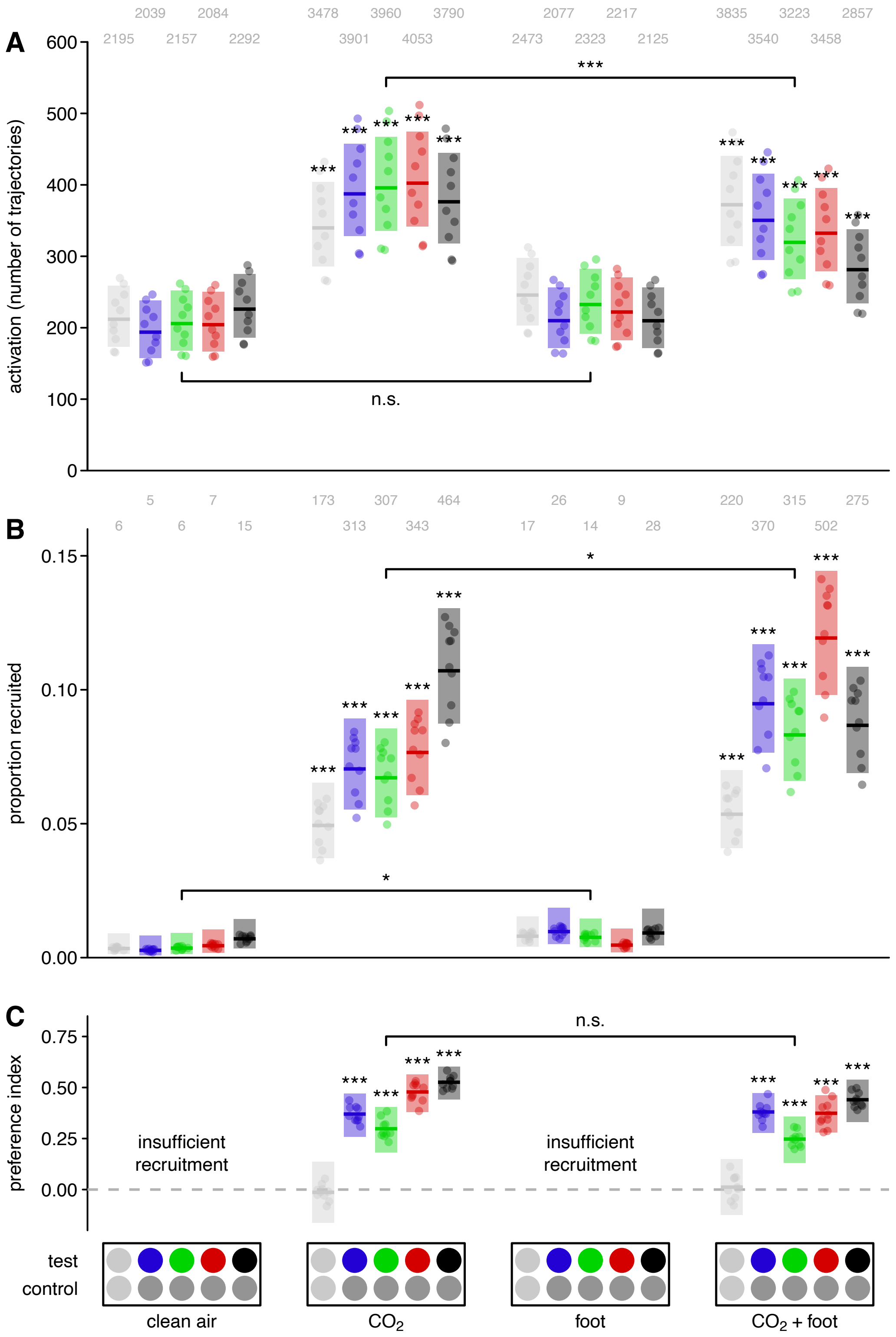

Fig. S5. Effect of foot odor on mosquito behavior. (A) Mosquito activation, as characterized by the number of mosquito trajectories recorded during each stimulus period where the plume consisted of clean air alone, 10% CO_2, 10% of the air passing through an odor jar containing a source of human foot odor, and the combination of CO_2 and foot odor. Asterisks above the boxes here indicate a statistical difference in the number of trajectories as compared with the stimulus period with paired neutral gray stimuli and clean air alone (leftmost box). When CO_2 was present, we found foot odor caused a significant decrease in the number of trajectories (significance bracket, *a priori* contrast, z = 3.34, P = 0.0008). Numbers above each boxplot indicate the total number of trajectories over 10 bioassay runs. (B) The proportion of trajectories recruited to either the test or control visual stimuli under the same odor conditions listed above. Asterisks above the boxes denote a statistically significant difference from the recruitment to paired neutral gray stimuli with clean air alone (leftmost box). We found a small, statistically significant, increase in the recruitment between clean air alone and foot odor (significance bracket, *a priori* contrast, z = -2.42, P = 0.0153), and a significant increase in the recruitment between CO_2 alone and the combination of and CO_2 and foot odor (significance bracket, *a priori* contrast, z = -2.48 P = 0.0129). Numbers between panels indicate the number of recruited trajectories over 10 bioassay runs. (C) The preference index of mosquitos in the wind tunnel responding to visual stimuli of various colors under the same odor conditions listed above. Significance stars above the boxes here indicate a difference from a preference index of 0.00. We found no evidence that foot odor in the plume influenced the visual preference of responding mosquitos, at least among this limited set of visual stimuli (significance bracket, likelihood-ratio test, χ^2 = 4.82 df = 5 P = 0.44). Test stimuli from left to right: neutral gray (light gray circles) at an intensity matching the fabric background, blue (450 nm), green (527 nm), red (621 nm) LEDs at matching isoquantal intensities, and unilluminated black tulle targets (black circles). Control stimuli: neutral or mid gray. Boxplots are the mean (line) with 95% confidence interval (shaded area), with points representing model predictions for each replicate bioassay run. Asterisks denote statistical differences: n.s. > 0.05, *P < 0.05, **P < 0.01, ***P < 0.001

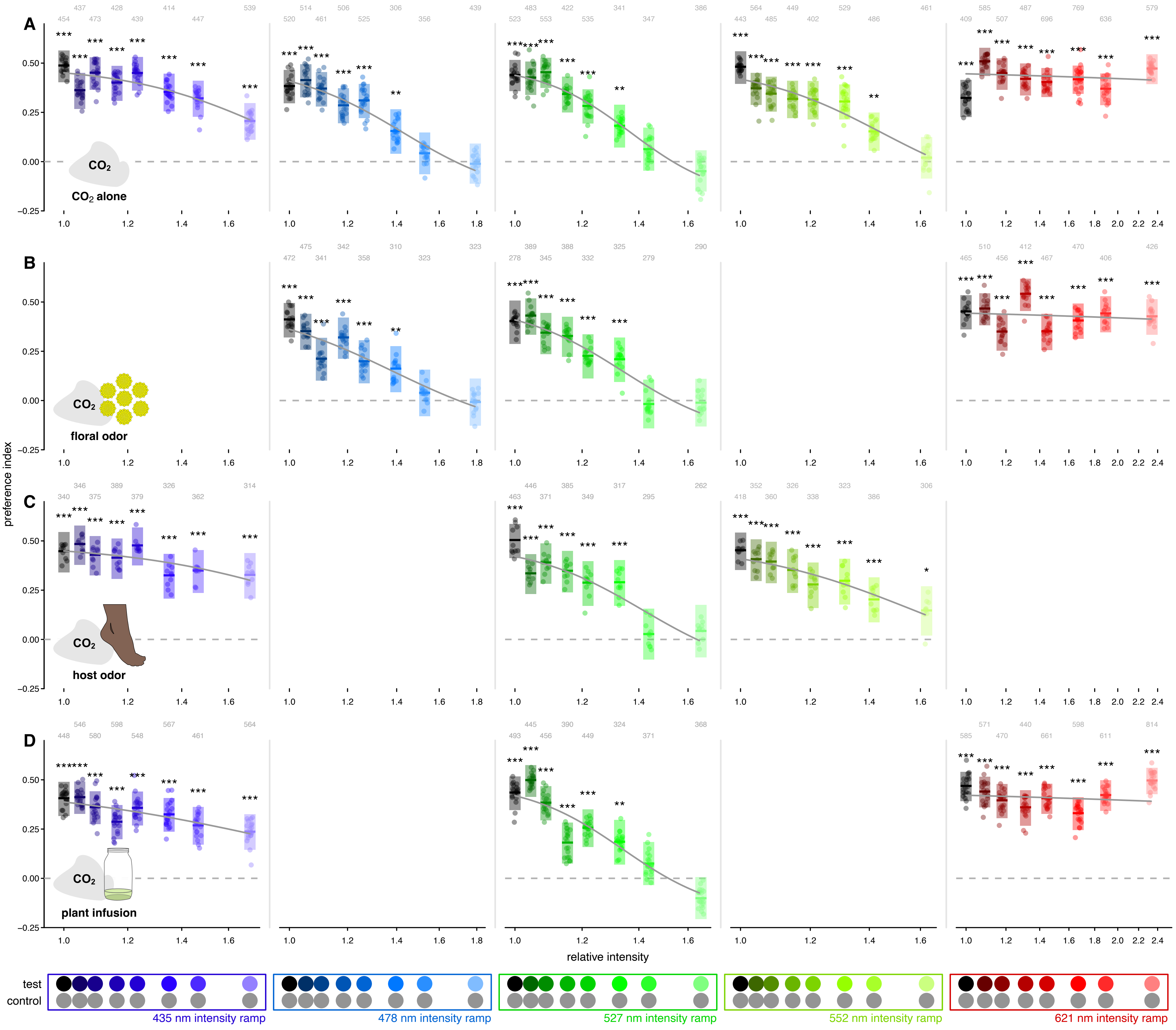

Fig. S6. Effect of odor and stimulus intensity on mosquito visual preference. Proportion of trajectories recruited to either the test or control visual stimulus in the presence of a 10% CO_2 plume paired with (A) no odor, (B) tansy (*T. vulgare*) floral odor, (C) human foot odor, and (D) the odor of an alfalfa infusion. We investigated the effect of intensity at a selection of wavelengths covering the visible range and focusing on spectral ranges where we observed odor shifts in spectral preferences in the spectral sweep experiments. The intensities on the x-axes are measured relative to the unilluminated black tulle targets, which were common among all of the intensity ramps, and non-zero due to the ambient illumination. Test stimuli: 435 nm, 478 nm, 527 nm, 552 nm, and 621 nm LED intensity ramps ranging in intensity from 0.0 to 3.0 times the isoquantal intensity used in the spectral sweep experiments. Control stimuli: mid gray. Boxplots are the mean (line) with 95% confidence interval (shaded area), with points representing model predictions for each replicate bioassay run. Numbers above each boxplot indicate the number of recruited trajectories over 20, 16, 10 and 20 replicate bioassay runs respectively. Gray lines show a sigmoid fitted to the preference data. Asterisks above the boxes denote a statistically significant difference from a preference index of 0.00. Asterisks denote statistical differences: *$P < 0.05$, **$P < 0.01$, ***$P < 0.001$
